# Investigation of Residue Dissipation of Fluxapyroxad and Its Metabolites in Chinese Cabbage and Spring Scallion Using Different Application Methods

**DOI:** 10.3390/plants13172448

**Published:** 2024-09-01

**Authors:** Ji Won Lee, Jin-Seong Kim, Ji Hyun Park, Hyun Ho Noh, Min Seok Oh, Jin-Hyo Kim, Kyeong-Ae Son

**Affiliations:** 1 Residual Agrochemical Evaluation Division, National Institute of Agricultural Science, RDA, Wanju 55365, Republic of Korea; jwlee0928@korea.kr (J.W.L.);; 2 Division of Applied Life Science, Institute of Agriculture and Life Science (IALS), Gyeongsang National University, Jinju 52828, Republic of Korea

**Keywords:** Chinese cabbage, dilution effect, fluxapyroxad, residue, systemic treatment, scallion

## Abstract

Fluxapyroxad, a persistent fungicide in soil, was investigated for differences in residue dissipation in Chinese cabbage and spring scallion through the application methods of soil, foliar, and systemic treatment. Soil application of 0.4% granule fluxapyroxad resulted in residues up to 0.09 mg kg^−1^ in the scallion, while it did not contribute to the residues in the harvested cabbage. The 50% dissipation time (DT50) of fluxapyroxad in the scallion was 6.8 days. The residues from systemic treatment were highly correlated with foliar application in both the cabbage and the scallion, and the initial residue and DT50 values were similar for foliar and systemic treatments. In comparing the residues from the systemic treatments between the two crops, the initial residue was 3.11 and 0.22 mg kg^−1^ in the cabbage and the scallion after the systemic treatment, respectively. The DT50 values were 2.6 and 12.2 days in the cabbage and the scallion, respectively. The theoretical dilution effect due to crop growth was higher for the cabbage (4-fold) than for the scallion (1.2-fold), and the half-lives of fluxapyroxad without considering the dilution effect were 6.4 days in the cabbage and 17.8 days in the scallion. Thus, the residue difference was drastically reduced after 14 days from the last treatment.

## 1. Introduction

Climate change affects the crop cultivation environment, and it changes the outbreak patterns of pests in crops [1,2,3,4]. Accordingly, pest control is becoming more challenging, and pesticides are needed to control emerging pests to maintain agricultural productivity in conventional agricultural practice, while the available active ingredients (AI) for pest control are limited [5,6,7,8,9]. To solve this problem, an AI has been evaluated for its novel biological efficacy against a range of pests, and these would be registered for use in the agricultural field [10,11,12,13]. However, when a single ingredient can be used for multiple purposes on a variety of crops, the residue pattern in the crop may change depending on the treatments that can be applied to soil, foliar, or both (systemic treatment) [14,15,16,17,18,19,20]. More specifically, systemic treatment could result in a different residue pattern and might be expected to produce a higher residue than a single treatment. Thus, the residue risk should be re-evaluated by considering the theoretical maximum dietary intake (TMDI) upon registration of a pesticide that may be used as a systemic treatment [19,20,21,22,23]. However, residue assessments conducted for registration were typically on individual treatment rather than systemic treatment, and the maximum residue limit (MRL) in crops was determined [20,24,25,26,27,28].

**Figure 1:**
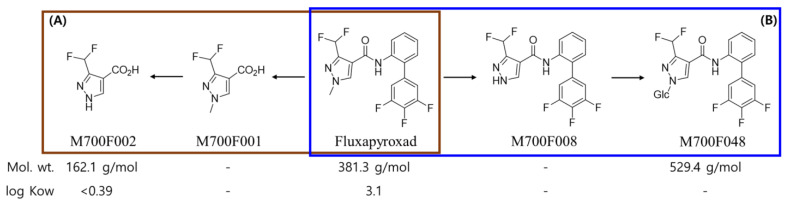
The proposed metabolic pathway for fluxapyroxad in soil (**A**, red box) and crops (**B**, blue box) [34].

Fluxapyroxad (3-(difluoromethyl)-1-methyl-N-(3′, 4′, 5′-trifluorobiphenyl-2-yl)pyrazole-4-carboxamide) is a carboxamide fungicide developed by BASF in 2012, and it is a systemic fungicide that controls pathogens by inhibiting succinic acid dehydrogenase [29,30,31]. It has been used worldwide for the control of fungal diseases in cereals, pulses, oilseeds, fruits, and vegetables. Regarding residue assessment, fluxapyroxad produced two main residual metabolites: M700F048 (3–(difluoromethyl)–1–(ß–D–glucopyranosyl)–N–(3′, 4′, 5′–triflurobipheny–2–yl)–1H–pyrzaole–4–carboxamide), which was a residual metabolite in crops, and M700F002 (3-(difluoromethyl)-1H-pyrazole-4-carboxylic acid), which was a residual metabolite in soil (Figure 1) [29,31,32]. Thus, pesticide risk assessment for fluxapyroxad was performed by summing the parent compound and its two metabolites in the Republic of Korea [20,33].

The 50% dissipation time (DT50) of fluxapyroxad was reported to be 183–1000 days [35,36,37], indicating its persistence in the soil environment, whereas the DT50 in crops (9.4–36.5 days) was significantly shorter than that in soil [38,39]. However, the residue in the soil environment could be transferred to crops, and this should be considered in assessing dietary exposure risks [19,38,40]. Therefore, systemic treatment combined with soil and foliar treatments during a single-crop cultivation would affect the residues in the harvested crop. However, this aspect might not be considered in detail during the registration step for minor crops. Thus, it is essential to evaluate the impact of the systemic treatment on crop residues to ensure compliance with Good Agricultural Practice (GAP). This evaluation was conducted to examine the dissipation and reduction of fluxapyroxad residues resulting from different treatments, including the dilution effect due to crop growth, in two vegetables with different growth rates: Chinese cabbage and spring scallion.

## 2. Results and Discussion

### 2.1. Method Validation for the Quantitative Analysis of Fluxapyroxad and Its Metabolites

The linearities (R^2^) of the matrix-matched calibration curves for fluxapyroxad and its two metabolites, M700F048 and M700F002, were >0.99, and the limit of quantification (LOQ) was 0.01 mg/kg for fluxapyroxad and M700F048 and 0.02 mg/kg for M700F002.

Recovery tests for each analyte were conducted at the LOQ and at 10× LOQ. The results showed recovery rates of 90.2–112.7% with a coefficient of variation (CV) of 1.0–6.4% for the cabbage and recovery rates of 84.0–104.3% with a CV of 0.3–11.2% for the scallion. The detailed information is presented in Table 1.

### 2.2. Effect of Systemic Application on Fluxapyroxad Residue in Chinese Cabbage

**Figure 2:**
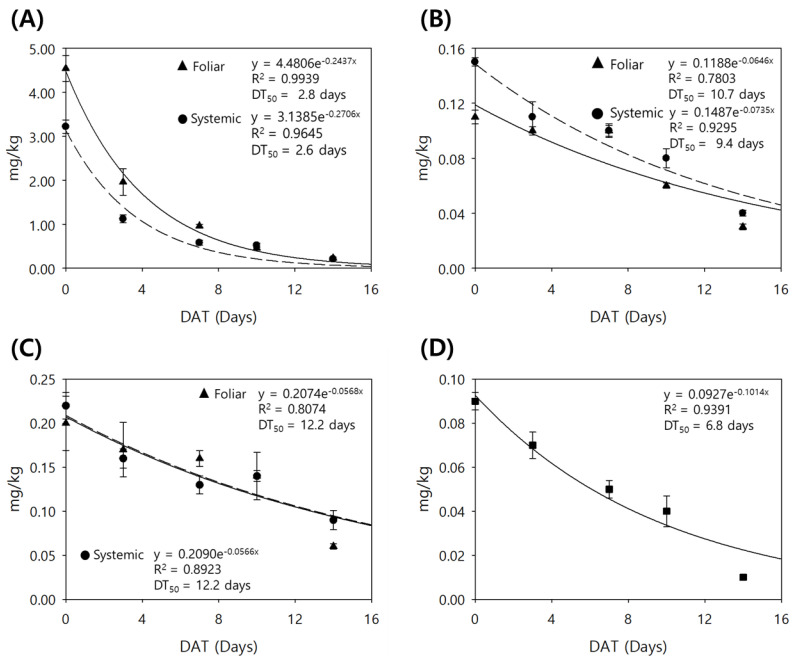
Dissipation of the total fluxapyroxad (**A**) and the metabolite M700F048 (**B**) in Chinese cabbage after foliar and systemic treatments, and the total fluxapyroxad in scallion after foliar and systemic treatments (**C**) and after soil treatment (**D**).

Fluxapyroxad residues were compared with three different fungicide applications: soil-only, foliar-only, and systemic (both soil and foliar) applications. The crop residues in each treatment decreased over time, from 0.01 mg kg^−1^ to below the LOQ in the soil treatment, from 4.54 to 0.23 mg kg^−1^ in the foliar treatment, and from 3.22 to 0.21 mg kg^−1^ in the systematic treatment (Table 2). The DT50 was estimated to be 2.6–2.8 days in foliar and systemic treatments (Figure 2). From the results, the crop residue contribution by the soil treatment was limited, considering the currently established maximum residue limit (MRL, 3.0 mg kg^−1^) in Chinese cabbage in South Korea [41]. Thus, the foliar application determined the residue in the systemic application, and the residue risk assessment from the foliar application would be sufficient to consider the residue from the systemic application.

### 2.3. Effect of Systemic Application on Fluxapyroxad Residue in Spring Scallion

Fluxapyroxad residues in scallion decreased over time from 0.09 to 0.01 mg kg^−1^ in the soil treatment, from 0.20 to 0.06 mg kg^−1^ in the foliar treatment, and from 0.22 to 0.09 mg kg^−1^ in the systemic treatment (Table 2). The DT50 was 6.8 days in the soil treatment, which was shorter than the foliar and systemic applications (12.2 days) (Figure 2). Although the soil treatment significantly contributed to the residue in the scallion, the residual concentration in the systemic treatment was determined by the foliar treatment. The result showed that the residue did not exceed the current MRL in the systemic treatment.

In addition, the initial residue in the scallion was 15–22-fold lower than that in the cabbage due to the difference in surface area between the two crops. Furthermore, the DT50 was 4-fold greater in the scallion compared to the cabbage, which can be attributed to the different dilution effects resulting from crop growth.

### 2.4. Crop Dilution and Degradation Effect on Fluxapyroxad Residue

The dilution effect of crop growth on fluxapyroxad residues was estimated from crop weight at harvest. The weight of each harvested crop and the theoretical diluted residue (TDR) without decomposition and removal, based on the initial residue, are presented in Table 3. The TDRs decreased due to crop growth in both Chinese cabbage and scallion, from 3.22 to 0.77 mg kg^−1^ and from 0.22 to 0.18 mg kg^−1^, respectively. Thus, the theoretical dilution effect by crop growth was higher for Chinese cabbage (4-fold) than for spring scallion (1.2-fold). Similarly, the detected residues in Chinese cabbage and scallion decreased from 3.22 to 0.21 mg kg^−1^ and from 0.22 to 0.09 mg kg^−1^, respectively.

**Figure 3:**
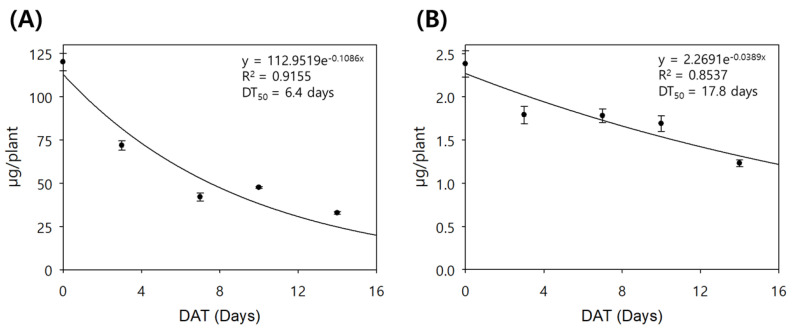
The estimated half-life of the total residue without dilution effect in the cabbage (**A**) and the scallion (**B**).

The detected residues were lower than the TDRs in both crops, and the residue differences could be caused by the dissipation amount due to decomposition, biological metabolism, evaporation, etc. [42]. The fluxapyroxad residue amount decreased from 120.1 to 32.8 mg plant^−1^ over time in the cabbage and from 2.38 to 1.23 mg plant^−1^ in the scallion, respectively. The dissipation ratios of the initial amount were 72.7% in the cabbage and 48.2% in the scallion after two weeks of cultivation. Finally, the half-lives of fluxapyroxad without the dilution effect were 6.4 days in the cabbage and 17.8 days in the scallion (Figure 3). From the results, fluxapyroxad was eliminated approximately 3-fold faster in the cabbage than in the scallion, and the dilution effect from crop growth reduced the DT50 in the cabbage by 3-fold, whereas it had a slight impact on the DT50 in the scallion. Consequently, although the initial residue of fluxapyroxad in the cabbage was approximately 15 times higher than that in the scallion, the residue at harvest 14 days after the final treatment was reduced to twice the level.

## 3. Materials and Methods

### 3.1. Standards and Reagents

Fluxapyroxad analytical standard was purchased from HPC Standards GmbH (Borsdorf, Germany). Metabolites (M700F048 and M700F002) of fluxapyroxad were obtained from BASF Co., Ltd. (Ludwigshafen, Germany). Acetonitrile (ACN), acetone, methanol, and water were used as high-performance liquid chromatography (HPLC) grade solvents (Burdick & Jackson^TM^, Honeywell International Inc., Charlotte, NC, USA). Ammonium formate and formic acid were purchased from Kanto Chemical Co. Inc. (Tokyo, Japan). Formic acid was obtained from Merck & Co., Inc. (Darmstadt, Germany). Sample extraction and purification were performed using a QuEChERS extraction kit (4 g of magnesium sulfate, MgSO_4_; 1g of sodium chloride, NaCl; 1 g of sodium citrate, Na_3_Citrate·2H_2_O; and 0.5 g of Na_2_HCitrate·1.5H_2_O) (Phenomenex Inc., Torrance, CA, USA) and EN method d-SPE (25 mg of primary secondary amine and 150 mg of MgSO_4_) (Agilent Technologies, Inc. Santa Clara, CA, USA).

### 3.2. Field Experiment and Fungicide Application

The seeds for Chinese cabbage (Haemaji-Eotgari) and spring scallion (Wangkwan-Hukkum-Kangoypa) were purchased from Mirae Seed Co. (Pohang, Republic of Korea). The fungicide application was carried out following the manual for safe pesticide application in the Korean GAP guideline [13,43]. Field experiments were conducted from March to May in a greenhouse in Jinju, Republic of Korea. The fungicide was applied at a rate of 6 kg/1000 m^2^ as 0.4% of GR-type fluxapyroxad (Nonghyup Chemical Co., Ltd., Seongnam, Republic of Korea) to the soil before seedlings were planted for the soil treatment. Additionally, 15.3% of suspension concentrate (SC)-type fluxapyroxad (Nonghyup Chemical Co., Ltd., Seongnam, Republic of Korea) was applied as a 4000-fold diluted solution to the crop foliar at 4.6 g AI/ 1000 m^2^ per application, with three applications at 7-day intervals. The fungicide application was divided into three groups: granule-only, foliar-only, and a combination of both treatments. Each treatment group size was 12 m^2^ with three replications. Chinese cabbage and spring scallion were harvested at intervals of 3–4 days, weighing more than 1.0 kg, at 31 and 42 days after seedlings were planted, respectively.

### 3.3. Sample Preparation for Residue Analysis of Fluxapyroxad

The harvested samples were chopped and pre-cooled at −20 °C for a day. Then, they were homogenized with dry ice. The samples were stored at −20 °C until residue analysis was conducted. A total of 10 mL of acetonitrile containing 0.2% formic acid was added to 10 g of the sample, and the QuEChERS extraction kit was added. Then, the mixture was vortexed and extracted at 200 rpm for 30 min (Daihan Large Digital Reciprocating Shaker, Daihan Scientific Co. Ltd., Wonju, Republic of Korea). The extract was subjected to centrifugation at 4000 rpm for 10 min (LABOGENE 1580R, LabogenTM, Bio-Medical Science Co., Ltd., Seoul, Korea), after which 1 mL of the supernatant was transferred to an e-tube containing the EN method d-SPE. This was followed by further vortexing and centrifugation at 12,000 rpm for 1 min. The supernatant was filtered with a 0.22 μm syringe filter (Advantec Co. Ltd., Toyo Roshi Kaisha, Ltd., Tokyo, Japan). The filtrate was instrumentally analyzed for fluxapyroxad and its two metabolites using an LC-triple quadrupole mass spectrometer (MS/MS) (Shimadzu Co. Ltd., Kyoto, Japan). The instrumental analysis conditions are described in Table 4.

### 3.4. Validation of Analytical Method

The validation of the analytical method was performed with reference to SANTE/2020/12830 and RDA criteria 2023 [44,45,46]. Various concentrations of standard solutions were analyzed by LC-MS/MS to determine the limit of quantitation (LOQ), defined as the lowest concentration meeting a signal-to-noise ratio (S/N) ≥ 10. A recovery test was conducted in triplicate at both the LOQ and 10× LOQ levels.

### 3.5. Total Fluxapyroxad

The total fluxapyroxad residue was calculated as the sum of fluxapyroxad and its two metabolite residues using a conversion factor derived from the molecular weight ratios [20,33]. The calculation equation is described as follows:

Total fluxapyroxad (mg kg^−1^) = Fluxapyroxad + (M700F002 × 2.35^a^) + (M700F048 × 0.72^b^) (1)

^a^ $2.35=\frac{381.3(molecular\;weight\;of\;fluxapyroxad)}{162.1\left(molecular\;weight\;ofM700F002\right)}$; ^b^ $0.72=\frac{381.3(molecular\;weight\;of\;fluxapyroxad)}{529.4\;(molecular\;weight\;ofM700F048)}$.

### 3.6. Crop Growth Dilution Effect

The dilution effect of the residue concentrations due to crop growth was estimated using the theoretical diluted residue (TDR) from the initial residue amount, and it was compared to the residue dissipation during crop cultivation. The equation for TDR was adapted from Choung et al. [18,47] and is described as follows:

(2) $$TDR\;(mg/kg)=\frac{\left[Initial\;residue(mg/kg)\times Initial\;crop\;weight\;(kg/plant)\right]}{Harvested\;crop\;weight\;(kg/plant)}$$

The dissipation ratio of fluxapyroxad was calculated using the initial residue amount and the detected residue amount. The equation is described as follows:

(3) $$\mathrm{Dissipation}\;\mathrm{ratio}\;(\%)=[1-\frac{\left[Residue\;amount(\mu g)\right]}{\left[Initial\;residue\;amount\;(\mu g)\right]}]\times 100$$

### 3.7. Statistical Analysis

To compare the weight changes of the crops and the temporal residue levels of fluxapyroxad across different treatments, a one-way analysis of variance (ANOVA) was performed using SPSS Statistics (ver. 12, IBM Corporation, Armonk, NY, USA). Significance was tested using Duncan’s multiple range test (DMRT) at the *p* < 0.05 level.

## 4. Conclusions

Soil application of 0.4% GR resulted in residues of up to 0.09 mg kg^−1^ in the scallion, while it did not contribute to the residues in the harvested Chinese cabbage. The residues resulting from systemic treatment were closely correlated with foliar application in both Chinese cabbage and spring scallion, and the DT50 values were similar for foliar and systemic treatments. In comparing the residues from the systemic treatments between the two crops, the initial residue level was significantly higher in Chinese cabbage, while the DT50 was lower in the cabbage due to a higher dilution effect from crop growth and a faster degradation rate. As a result, the residue difference was drastically reduced after 14 days from the last treatment, and residue levels were below the established MRLs.

## Figures and Tables

**Table 1:** Information on the quantitative analysis method validation for fluxapyroxad and its metabolites.

Matrix	Compound	R^2^	LOQ (mg kg^−1^)	Mean Recoveries ± SD(%, *n* = 3)			
				0.01 mg kg^−1^	CV(%)	0.1 mg kg^−1^	CV(%)
Chinese cabbage	Fluxapyroxad	0.9995	0.01	99.2 ± 3.9	2.6	109.6 ± 0.9	1.0
	M700F048	0.9992	0.01	90.2 ±4.1	6.4	112.7 ± 1.8	1.7
	M700F002	0.9993	0.02	104.0 ± 2.5	1.1	97.8 ± 4.6	1.5
Scallion	Fluxapyroxad	0.9998	0.01	84.7 ± 2.0	2.7	104.3 ± 1.7	2.3
	M700F048	0.9998	0.01	95.7 ± 3.7	3.3	99.8 ± 1.2	1.6
	M700F002	0.9998	0.02	84.0 ± 8.1	11.2	96.1 ± 3.2	0.3

**Table 2:** Fluxapyroxad residue (mg kg^−1^) in the cabbage and the scallion after soil-only, foliar-only, and systemic treatments.

Compounds	DAT ^a^ (days)	Detected Residue (mg kg^−1^) (CV)					
		Chinese Cabbage			Spring Scallion		
		Soil	Foliar	Systemic	Soil	Foliar	Systemic
Fluxapyroxad	0	<0.01	4.46 (6.5)	3.11 (4.7)	0.09 (4.7)	0.20 (15.3)	0.22 (6.8)
	3	<0.01	1.89 (15.9)	1.03 (8.2)	0.07 (8.2)	0.17 (18.5)	0.16 (6.7)
	7	<0.01	0.89 (4.9)	0.51 (11.8)	0.05 (8.2)	0.16 (5.4)	0.13 (7.6)
	10	<0.01	0.41 (6.5)	0.46 (9.1)	0.04 (16.7)	0.14 (18.5)	0.14 (4.6)
	14	<0.01	0.21 (9.8)	0.18 (3.5)	0.01 (9.1)	0.06 (5.6)	0.09 (13.4)
M700F048	0	0.02 (16.8)	0.11 (4.3)	0.15 (2.2)	<0.01	<0.01	<0.01
	3	<0.01	0.10 (3.3)	0.11 (9.1)	<0.01	<0.01	<0.01
	7	<0.01	0.10 (4.4)	0.10 (5.3)	<0.01	<0.01	<0.01
	10	<0.01	0.06 (3.6)	0.08 (8.0)	<0.01	<0.01	<0.01
	14	<0.01	0.03 (7.0)	0.04 (4.1)	<0.01	<0.01	<0.01
M700F002	0	<0.02	<0.02	<0.02	<0.02	<0.02	<0.02
	3	<0.02	<0.02	<0.02	<0.02	<0.02	<0.02
	7	<0.02	<0.02	<0.02	<0.02	<0.02	<0.02
	10	<0.02	<0.02	<0.02	<0.02	<0.02	<0.02
	14	<0.02	<0.02	<0.02	<0.02	<0.02	<0.02
Total Fluxapyroxad	0	0.01 (17.3)	4.54 (6.4)	3.22 (4.6)	0.09 (4.7)	0.20 (15.3)	0.22 (6.8)
	3	<0.01	1.96 (15.3)	1.12 (8.2)	0.07 (8.2)	0.17 (18.5)	0.16 (6.7)
	7	<0.01	0.96 (4.6)	0.58 (10.9)	0.05 (8.2)	0.16 (5.4)	0.13 (7.6)
	10	<0.01	0.46 (6.0)	0.52 (9.0)	0.04 (16.7)	0.14 (18.5)	0.14 (4.6)
	14	<0.01	0.23 (9.5)	0.21 (3.2)	0.01 (9.1)	0.06 (5.6)	0.09 (13.4)

^a^ DAT means days after the final treatment.

**Table 3:** Crop weight changes, the estimated TDR, and fluxapyroxad residue amount in the cabbage and the scallion.

Crop	DAT ^a^	Crop Weight (g_fw_ plant^−1^) ^b^	Detected Residue (mg kg^−1^, CV)	TDR (mg kg^−1^)	Residue Amount (μg plant^−1^)	Dissipation Ratio ^c^
Chinese cabbage	0	37.3a	3.22, 4.6%	3.22	120.1	-
	3	64.1b	1.12, 8.2%	1.87	71.8	40.2%
	7	72.4bc	0.58, 10.9%	1.66	42.0	65.0%
	10	91.4c	0.52, 9.0%	1.31	47.5	60.4%
	14	156.2d	0.21, 3.2%	0.77	32.8	72.7%
Spring scallion	0	10.8a	0.22, 6.8%	0.22	2.38	
	3	11.2a	0.16, 6.7%	0.21	1.79	24.6%
	7	13.7a	0.13, 7.6%	0.17	1.78	25.0%
	10	13.2a	0.14, 4.6%	0.18	1.69	28.9%
	14	13.1a	0.09, 13.4%	0.18	1.23	48.2%

^a^ DAT means days after the final foliar treatment; ^b^ the letters were the results of Duncan’s multiple range test (DMRT, *p* <0.05); ^c^ dissipation ratio was calculated from the initial residue amount with dissipation amount.

**Table 4:** LC-MS/MS instrumental analysis conditions for fluxapyroxad and its metabolites.

Instrument	Shimadzu Triple Quadrupole LCMS-8050							
Analytes	Fluxapyroxad, M700F048				M700F002			
Column	Kinetex phenyl-hexyl (100 × 2.1 mm, 2.6 µm)				Hypercarb (100 × 2.1 mm, 3 µm)			
Mobile phase A	0.1% formic acid in dH_2_O with 5 mM ammonium formate				0.1% formic acid in dH_2_O with 5 mM ammonium formate			
Mobile phase B	0.1% formic acid in ACN with 5 mM ammonium formate				0.1% formic acid in ACN			
Gradient	Time (min)		A (%)	B(%)	Time (min)		A (%)	B(%)
	0		85	15	0		70	30
	1.5		40	60	5		70	30
	8		10	90	5.5		0	100
	8.1		0	100	14		0	100
	15		0	100	14.5		70	30
Flow rate	0.2 mL/min				0.3 mL/min			
Injection volume	1 µL				5 µL			
ColumnTemp.	35 °C				35 °C			
Ionization mode	ESI positive							
Scan type	MRM mode							
Detection ion (*m/z*)	Analyte	Precursor	Quantitative (CE)	Qualitative (CE)	Analyte	Precursor	Quantitative (CE)	Qualitative (CE)
	Fluxapyroxad	381.7	362.1 (−17)	342.0 (−23)	M700F002	161.0	141.0 (−14)	116.9 (−14)
	M700F048	529.8	347.7 (−23)	367.8 (−23)				

## Data Availability

The data is contained within the manuscript.
